# I Do Help: Older Adults as Digital Media Support Providers for Their Peers

**DOI:** 10.1093/geroni/igaa057.1516

**Published:** 2020-12-16

**Authors:** Amanda Hunsaker, Minh Hao Nguyen, Jaelle Fuchs, Gökçe Karaoglu, Teodora Djukaric, Eszter Hargittai

**Affiliations:** University of Zurich, Zurich, Switzerland

## Abstract

Older adults include highly sophisticated digital media users among their numbers for whom diverse methods of using online technology are a daily occurrence. Given that some older adults are quite tech-savvy, it follows that they may also provide digital media support to others. This study examines technological support-giving abilities and experiences of older adults. We completed in-depth qualitative interviews with older adults (ages 59+) in Hungary, the Netherlands, Switzerland, and Turkey (N=63) exploring: (1) whether older adults give digital media support and reasons for (not) doing so; (2) how older adults provide support and to whom; and (3) experiences of providing support with IT security specifically. The research team conducted consensus meetings to identify themes and coding schemes, and memo writing to share findings and ensure reliability across and within coders. Overall, a large number of our participants reported giving digital media support, primarily within their social circles. The kinds of help provided highly varied from navigating the Internet to choosing devices and managing security settings. We also find mutual support – support given to each other – an important domain for how people in this age group share assistance for technical problems. While some participants did not provide help with digital technology because they lacked confidence to do so, others believed they could, but were never asked. That such a prominent amount of support providers exist in this age group implies that peer-led technical support approaches may be especially salient and effective in helping older adults use digital media.

